# Startle disease in Irish wolfhounds associated with a microdeletion in the glycine transporter GlyT2 gene

**DOI:** 10.1016/j.nbd.2011.03.010

**Published:** 2011-07

**Authors:** Jennifer L. Gill, Deborah Capper, Jean-François Vanbellinghen, Seo-Kyung Chung, Robert J. Higgins, Mark I. Rees, G. Diane Shelton, Robert J. Harvey

**Affiliations:** a Department of Pharmacology, The School of Pharmacy, 29–39 Brunswick Square, London WC1N 1AX, UK; b Halsey East Animal Clinic, 16057 NE Halsey Street, Portland, OR 97230, USA; c Secteur Commun de Biologie Moléculaire–Unilab-Lg, Centre Hospitalier Universitaire de Liège, 4000 Liège, Belgium; d Institute of Life Science, College of Medicine, Swansea University, Swansea SA2 8PP, UK; e Department of Pathology, Microbiology and Immunology, University of California, Davis, CA 95616, USA; f Department of Pathology, University of California, San Diego, La Jolla, CA 92093–0709, USA

**Keywords:** Glycine transporter, GlyT2, Hyperekplexia, Irish wolfhound, *SLC6A5*, Startle disease

## Abstract

Defects in glycinergic synaptic transmission in humans, cattle, and rodents result in an exaggerated startle reflex and hypertonia in response to either acoustic or tactile stimuli. Molecular genetic studies have determined that mutations in the genes encoding the postsynaptic glycine receptor (GlyR) α1 and β subunits (*GLRA1* and *GLRB*) and the presynaptic glycine transporter GlyT2 (*SLC6A5*) are the major cause of these disorders. Here, we report the first genetically confirmed canine cases of startle disease. A litter of seven Irish wolfhounds was identified in which two puppies developed muscle stiffness and tremor in response to handling. Although sequencing of *GLRA1* and *GLRB* did not reveal any pathogenic mutations, analysis of *SLC6A5* revealed a homozygous 4.2 kb microdeletion encompassing exons 2 and 3 in both affected animals. This results in the loss of part of the large cytoplasmic N-terminus and all subsequent transmembrane domains due to a frameshift. This genetic lesion was confirmed by defining the deletion breakpoint, Southern blotting, and multiplex ligation-dependent probe amplification (MLPA). This analysis enabled the development of a rapid genotyping test that revealed heterozygosity for the deletion in the dam and sire and three other siblings, confirming recessive inheritance. Wider testing of related animals has identified a total of 13 carriers of the *SLC6A5* deletion as well as non-carrier animals. These findings will inform future breeding strategies and enable a rational pharmacotherapy of this new canine disorder.

## Research Highlights

► Startle disease is caused by mutations in glycine receptor and transporter genes. ► We report the first genetically confirmed cases of canine startle disease. ► We found a novel microdeletion in *SLC6A5* encoding the glycine transporter GlyT2. ► We can now offer diagnostic testing of carriers and potentially treat this disorder. ► Deletion detection techniques should be applied in human startle disease genetics.

## Introduction

Hyperekplexia or startle disease affects newborn children and is characterized by noise- or touch-induced non-epileptic seizures that result in muscle stiffness and apnea (suspension of breathing). Although a rare orphan disease (defined as < 200,000 affected individuals worldwide), this disorder can have serious consequences, including brain damage and/or sudden infant death (Harvey et al., 2008). The primary cause of startle disease is defective inhibitory glycinergic transmission due to missense, nonsense, or frameshift mutations in the glycine receptor (GlyR) α1 gene (*GLRA1*) (Shiang et al., 1993, 1995; Chung et al., 2010), although large deletions of exons 1–7 have also been reported (Brune et al., 1996; Becker et al., 2006). Mutations are also found in the genes encoding the GlyR β subunit gene (*GLRB*; Rees et al., 2002) and the presynaptic glycine transporter GlyT2 (*SLC6A5*) (Rees et al., 2006; Eulenburg et al., 2006). Animal disorders similar to startle disease/hyperekplexia have been reported in cattle (Harper et al., 1986; Gundlach, 1990; Gundlach et al., 1988; Lummis et al., 1990; Pierce et al., 2001; Charlier et al., 2008; Gill et al., 2011), horses (Gundlach et al., 1993), and dogs (Fox et al., 1984), but only in certain cases has the underlying genetic lesion been definitively identified (Table 1). For example, a nonsense mutation (c.C156A, p.Y24X) in exon 2 of *GLRA1* causes inherited congenital myoclonus (ICM) in Poll Hereford calves (Pierce et al., 2001), while a missense mutation (c.T809C, p.L270P) in exon 4 of *SLC6A5* causes congenital muscular dystonia 2 (CMD2) in Belgian Blue cattle (Charlier et al., 2008; Gill et al., 2011). The identification of common causal genes links these animal disorders to human startle disease/hyperekplexia. Two other related disorders—inherited myoclonus in Peruvian Paso horses (Gundlach et al., 1993) and familial reflex myoclonus in Labrador retrievers (Fox et al., 1984)—are reminiscent of deficits in glycinergic transmission, although investigations into the underlying genetic causes are currently limited due to a lack of DNA samples from affected animals. These startle disorders should be distinguished from exercise-induced muscle hypertonicity disorders, such as episodic falling in Cavalier King Charles spaniels (Herrtage and Palmer, 1983).

Here, we report the discovery of startle disease in Irish wolfhounds from the United States and identify the causative mutation—a microdeletion in the gene encoding the presynaptic glycine transporter GlyT2. We also demonstrate how rapid genotyping tests can be used to confirm diagnosis, identify carriers, and guide future breeding strategy, thus avoiding further animal distress and premature death.

## Methods

### Histopathology and histochemistry

A full necropsy was performed on both affected puppies following humane euthanasia. Tissues including brain and spinal cord were processed into paraffin by standard procedures. In addition, specimens from the biceps femoris, vastus lateralis, and triceps brachii muscles were collected and frozen in isopentane pre-cooled in liquid nitrogen. Cryosections were cut (8 μm) and the following histochemical stains and reactions were performed: hematoxylin and eosin, modified Gomori trichrome, periodic acid Schiff, phosphorylase, esterase, ATPase reactions at pH of 9.8 and 4.3, nicotinamide adenine dinucleotide-tetrazolium reductase, succinic dehydrogenase, acid phosphatase, alkaline phosphatase, and oil red O (Dubowitz and Sewry, 2007).

### PCR and DNA sequencing

PCR was performed using 50 ng genomic DNA as template and AccuPrime *Pfx* SuperMix with exon-specific primers for *GLRA1*, *GLRB*, and *SLC6A5* (Table S1). PCR conditions were 35 cycles of 94 °C for 1 min, 60 °C for 1 min, and 68 °C for 2 min. PCR products were gel purified using a QIAQuick Gel Extraction Kit (QIAGEN) for Sanger DNA sequencing by DNA Sequencing and Services (MRC-PPU, College of Life Sciences, University of Dundee, Scotland; www.dnaseq.co.uk) using Applied Biosystems Big-Dye version 3.1 chemistry on an ABI 3730 automated capillary DNA sequencer. DNA sequences were analyzed using Sequencher (Gene Codes Corporation, Ann Arbor, USA).

### Southern blotting

Genomic DNA (2.5 μg) was digested with *Hin*dIII, separated on a 1% (wt./vol.) agarose gel and subjected to Southern blotting onto Hybond-N membrane (GE Healthcare, Little Chalfont, UK). The blot was probed using a *SLC6A5* exon 1 PCR amplicon radiolabeled using α[^^32^^P]dCTP and the Prime-It II Random Primer Labeling Kit (Agilent, Wokingham, UK). Hybridization was at 65 °C overnight in a buffer containing 6× SSC, 10% (wt./vol.) Dextran sulphate, 1% (wt./vol.) SDS, 5× Denhardt's Solution, and 100 μg/ml denatured herring sperm DNA. Final washes of the blot were at 0.1× SSC, 0.1% SDS at 65 °C for 30 min. Hybridizing fragments were revealed by exposure to X-ray film for 14 days.

### Multiplex ligation-dependent probe amplification (MLPA) analysis

We designed five MLPA probe pairs corresponding to the first five coding exons of the canine GlyT2 gene (*SLC6A5*; NC_006603 on chromosome 21; Table S2). Probes for exon 2 were positioned just inside intron 2, due to the high GC content (78%) of this exon. Criteria for MLPA probe design were as previously described (Schouten et al., 2002). Control probe pairs were designed to recognize an unrelated gene (CFTR: NC_006596) on chromosome 14. Probes generated amplification products ranging in size from 87 to 113 bp and had an annealing temperature higher than 70 °C as recommended in RAW Probe software (MRC-Holland, Amsterdam, The Netherlands) using standard MLPA conditions (Schouten et al., 2002). PCR products were analyzed on an ABI 3130xl capillary electrophoresis apparatus (Applied Biosystems, Lennik, Belgium). Normalization of *SLC6A5* specific probe signals was performed by dividing the values obtained by the combined signal of the control probes.

## Results

### Startle disease in Irish wolfhounds

**Fig. 1:**
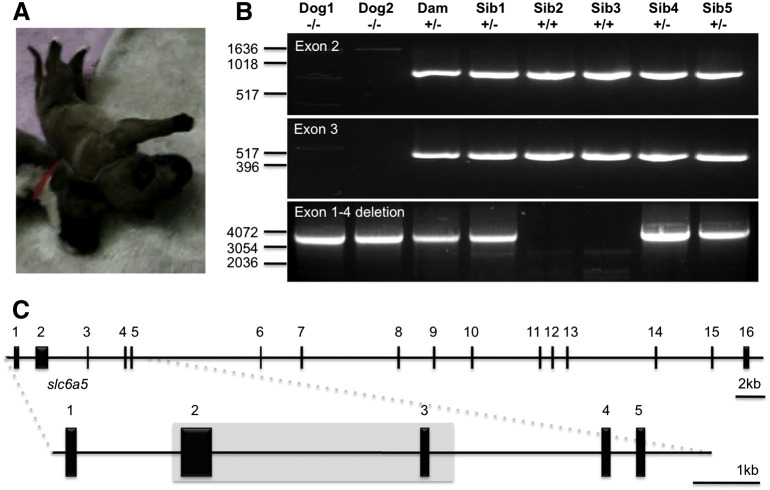
PCR assays reveal that a *SLC6A5* microdeletion is associated with startle disease in Irish wolfhounds. (A) Photograph of one of two affected puppies demonstrating lateral recumbency and an extended posture (see Video S1 online). (B) PCR panels for *SLC6A5* exon 2 (754 bp), exon 3 (513 bp), and a large genomic fragment encompassing exons 1–4 (target size, 8.2 kb). Note that exon 2 and exon 3 fragments fail to amplify from the affected dogs (samples and 1 and 2, −/−) but can be robustly amplified using genomic DNA from the dam and five other siblings. Although the 8.2 kb exon 1–4 fragment was not amplified from wild-type animals or carriers (due to the large size and GC-rich nature of the amplicon), a 4 kb PCR product was observed in both affected dogs (−/−), the dam, and three of the littermates (sibs 1, 4, and 5—all likely to have a +/− genotype). (C) Since exons 1 and 4–16 were amplified from affected animals, these results strongly suggest that a microdeletion encompassing *SLC6A5* exons 2 and 3 is the genetic cause of this startle disorder, with the two remaining animals (sibs 2 and 3) being homozygous for the wild-type allele (+/+).

Two Irish wolfhound puppies, one male and one female, were born with clinical signs similar to human hyperekplexia and other glycinergic disorders observed in cattle (Fig. 1A, Table 1, and Video S1 online). The puppies, whose five siblings all appeared normal, developed extensor rigidity and tremor beginning 5–7 days post-partum that was evoked by handling and ceased when the animals were relaxed or sleeping. The puppies were unable to stand and showed a typical rigid extended posture in all four limbs. Cyanosis was noted while the dogs were suckling or during tube feeding. Due to the progression of clinical signs, feeding difficulty, and development of lung congestion, the dogs were humanely euthanized at approximately 11 weeks of age and full necropsies were performed.

### Histopathology and histochemistry

Both puppies were small for their age with weights of 1446 and 1559 g, while the normal littermates (*n* = 5) averaged 2642 g, ranging from 2495 to 2778 g. The primary abnormality on gross examination of both animals was consolidated (neither floated in formalin) and hemorrhagic lungs, with the male puppy more severely affected than the female. The esophagus in each of the puppies was noticeably dilated, more so in the female than the male. No macroscopic abnormalities of the brain and spinal cord were noted. At the light microscopic level, moderate interstitial pneumonia was noted in both puppies. No significant pathological abnormalities were noted in the brain or spinal cord of either animal. All muscles appeared to have developed normally with myofiber size appropriate for the age of the puppies and a normal mosaic pattern of muscle fiber types. Intramuscular nerve branches were normal in appearance. There was no evidence of a primary muscle or nerve disease in any of the muscle specimens.

### Candidate gene analysis—*GLRA1*, *GLRB*, and *SLC6A5*

Given the similarity of clinical signs to startle disease, primers were designed to amplify exons and flanking splice donor, acceptor, and branch-point sites for the major causative genes, *GLRA1*, *GLRB*, and *SLC6A5*, deriving gene structures in silico using the UCSC Genome Browser (http://genome.ucsc.edu/). PCR amplification (for primers, see Table S1) using proofreading *Pfx* DNA polymerase and direct sequencing revealed surprisingly few exonic variants in *GLRA1* and *GLRB*. No SNPs were detected in *GLRA1*, and only two homozygous SNPs were detected in *GLRB*: T1380A (p.P438P, dbSNP: rs22406580) and c.G1226A causing a p.C385Y substitution in the mature GlyR β subunit M3–M4 intracellular loop. However, both these variants were also found in unaffected littermates, ruling out a pathogenic role. Variation was also absent from exons 1 and 4–16 of the *SLC6A5* gene. However, we consistently failed to amplify *SLC6A5* exons 2 and 3 with several different primer sets from genomic DNA isolated from the two affected dogs. Since control experiments revealed that these primer sets were functional in PCRs using template DNAs from the unaffected littermates and dam (Fig. 1), we suspected that a microdeletion encompassing exons 2 and 3 could explain this anomaly. To investigate this possibility, we used long-distance PCR to amplify the genomic region encompassing *SLC6A5* exons 1–4 generating a fragment predicted to be 8.2 kb in size. Although we failed to amplify this fragment from control DNAs (presumably due to the highly GC-rich exon 2) in the two affected dogs, the unaffected dam, and three of the unaffected littermates, we observed robust PCR products migrating at 4 kb, while the two remaining unaffected littermates had no PCR products in this size range. These results suggested that the two affected dogs were homozygous for a microdeletion of 4.2 kb, while the dam and littermates 1, 4, and 5 were heterozygous for this deletion. Littermates 2 and 3 would be predicted to be homozygous wild-type due to the absence of the 4 kb deletion fragment.

### Mapping the *SLC6A5* deletion breakpoint

**Fig. 2:**
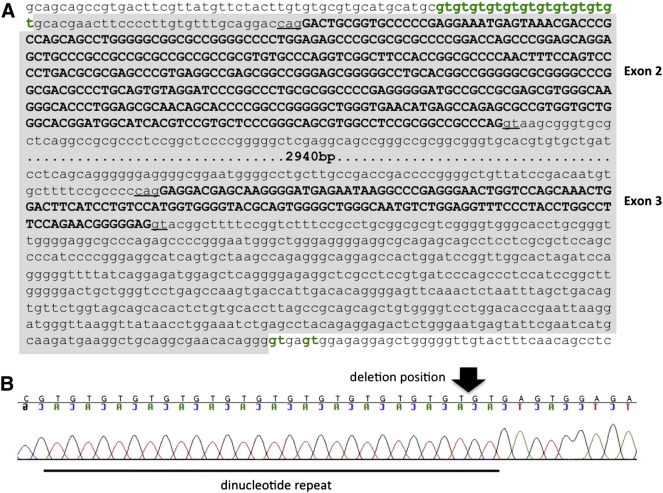
Sequencing of the *SLC6A5* exon 1–4 amplicon reveals a microdeletion encompassing exons 2 and 3. (A) DNA sequence of the *SLC6A5* deletion fragment from affected animals and the dam revealed a 4230 bp microdeletion, resulting in the loss of exons 2 and 3 encoding the GlyT2 N-terminus. (B) Breakpoint sequence with the position of the microdeletion indicated by an arrow. Note that the deletion is positioned just downstream of a dinucleotide (gt) repeat, and while the dog genome consensus sequence contains 11 copies of this dinucleotide repeat, all Irish wolfhounds genotyped in this study (wild-type, heterozygous, and mutant) had 14 copies of this repeat.

**Fig. 3:**
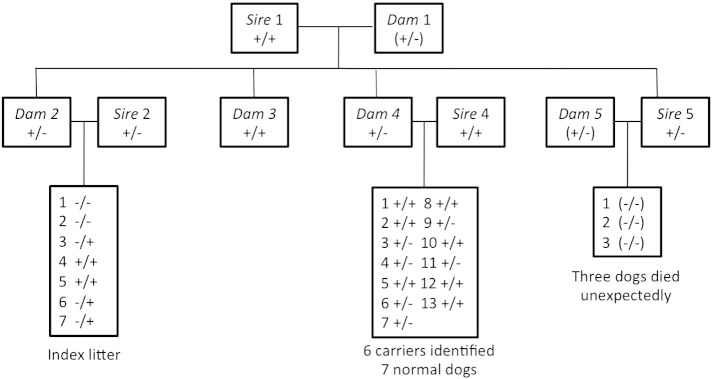
Combined pedigree analysis and genotyping reveals additional *SLC6A5* microdeletion carriers. Pedigree analysis combined with PCR genotyping of exon 2, 3, and microdeletion amplicons (see Fig. 1) allowed the identification of a total of 13 heterozygous carriers and 2 additional suspected carriers (dam 1 and dam 5). Notably, we were able to offer rapid diagnostic genotyping for the large litter from dam 4 and sire 4, allowing the owner to manage future breeding strategy. By distinguishing phenotypically identical carriers (+/−) from normal (+/+) animals, this disorder can effectively be eliminated from breeding stock in a single generation. It is also noteworthy that three offspring of sire 5 died unexpectedly, suggesting that dam 5 may also be a carrier of the *SLC6A5* microdeletion. For genotypes in brackets, DNA samples were not available and genotypes are suggested rather than categorically proven.

In order to confirm this deletion and determine the exact breakpoint in the deleted allele, we cloned and sequenced the 4 kb deletion fragment. This analysis revealed that the deletion spanned both exons 2 and 3, starting 32 bp upstream of exon 2 and ending 504 bp downstream of the end of exon 3 (Figs. 1 and 2). The presence of a dinucleotide (gt) repeat at the breakpoint is relevant since a common cause of microdeletions is slipped mispairing of direct repeat sequences in close proximity to one another. This can occur during DNA replication when the primer and template strands can transiently dissociate and reassociate in a misaligned configuration. If the primer strand which contains the newly synthesized first direct repeat dissociates from the template strand and misaligns by slipping forward at the second direct repeat, then continued DNA synthesis will result in the deletion of one of the direct repeats as well as the intervening sequence (Ball et al., 2005). Additional pedigree analysis and genotyping of DNA samples from related animals (Fig. 3) revealed a total of 13 carriers of the GlyT2 microdeletion—including the sire of the affected dogs—and two other likely carriers. Although DNA samples were not available for all animals in the pedigree, this provided a possible explanation for the unexplained deaths of three animals in another litter, where we confirmed that the sire was a carrier of the *SLC6A5* lesion (Fig. 3).

**Fig. 4:**
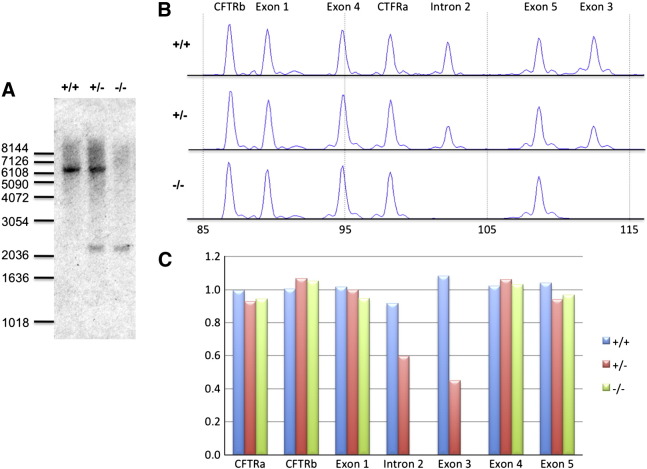
Confirmation of the *SLC6A5* deletion using Southern blotting and multiplex ligation-dependent probe amplification. (A) Southern blotting of genomic DNA digested with *Hin*dIII and probed with the exon 1 PCR amplicon is predicted to reveal a 6672 bp fragment in wild-type animals and a 2242 bp fragment in animals harboring the microdeletion. The observed sizes and banding patterns of DNA fragments hybridizing with the exon 1 probe are in good agreement with these values in wild-type (+/+), heterozygous (+/−), and homozygous affected (−/−) animals. (B, C) MLPA analysis also reveals robust detection of control probes (CFTRa and CFTRb), probes for *SLC6A5* exons 1, 4, and 5. However, signals for probes in intron 2 (just downstream of the GC rich exon 2) and exon 3 were reduced by 35%–60% in heterozygous (+/−) animals and abolished in homozygous animals (−/−) consistent with a loss of probe binding sites in genomic DNA.

The deletion of exons 2 and 3 results in the loss of part of the large cytoplasmic N-terminus, thought to be involved in the trafficking of GlyT2 to synaptic sites, and a loss of all subsequent transmembrane domains via a frameshift. We also confirmed the presence of the microdeletion using two additional methods—Southern blotting (Southern, 2006) using a probe derived from exon 1 and multiplex ligation-dependent probe amplification (MLPA; Schouten et al., 2002) using probe sequences in exons 1, 3, 4, and 5, and intron 2 (Table S2). Both methods confirmed the loss of exons 2 and 3 in heterozygotes and homozygous affected animals (Fig. 4).

## Discussion

This study describes the clinical signs and pathological changes associated with a new canine disorder: startle disease in Irish wolfhounds. In addition, we report the underlying genetic cause—a microdeletion in the GlyT2 gene *SLC6A5*. Consistent with previous reports of GlyT2 mutations in human hyperekplexia (Rees et al., 2006; Eulenburg et al., 2006) and bovine congenital muscular dystonia type 2 (Charlier et al., 2008; Gill et al., 2011), this canine disorder exhibited an autosomal recessive mode of inheritance, consistent with a loss of function of GlyT2. However, it is noteworthy that this study represents the first report of a *SLC6A5* microdeletion in any species, suggesting that copy number variations in the GlyT2 gene should be considered a risk factor for startle disorders.

The clinical phenotype produced as a result of the microdeletion identified in the affected wolfhounds most likely results from a loss of synaptic glycine since the major role of GlyT2 is to maintain a high presynaptic pool of glycine for inhibitory neurotransmission (Gomeza et al., 2003; Rousseau et al., 2008). In GlyT2 knockout mice, this results in the development of an acute motor disorder in the second postnatal week, characterized by rigidity, tremor, and an impaired righting reflex that is followed shortly after by death (Gomeza et al., 2003). Similarly, cattle with CMD2 caused by a missense mutation (p.L270P) in the GlyT2 gene suffer severe episodes of muscle rigidity and tremor upon acoustic or tactile stimulation and usually die shortly after birth due to respiratory difficulties (Charlier et al., 2008; Gill et al., 2011). This early neonatal lethality might be explained by the fact that defective presynaptic glycine uptake is predicted to affect the function of all GlyR subtypes. In particular, GlyRs containing the α3 subunit have recently been shown to play a role in rhythmic breathing (Manzke et al., 2010) which may explain the breathing difficulties seen in mice, cattle, and dogs with inactivating *SLC6A5* mutations. Given the cyanosis that developed during feeding and the pneumonia observed in both animals which may have been caused or exacerbated by compromised lung clearance, both puppies would have likely died if they had not been humanely euthanized. However, no obvious lesions in the brain, spinal cord, and muscle were observed, suggesting that pharmacotherapy might have been effective. In this regard, it is noteworthy that humans with mutations in the GlyT2 gene also present with neonatal touch and noise induced muscle stiffness and apnea episodes (Rees et al., 2006; Eulenburg et al., 2006). These symptoms respond to therapy with the benzodiazepine clonazepam, which potentiates inhibitory GABAergic transmission and often improve in the first year of life (Rees et al., 2006; Eulenburg et al., 2006; Bakker et al., 2009). These differences suggest not only that compensatory mechanisms may exist in humans that do not operate in other species but also that early intervention with clonazepam could treat the clinical signs and improve survival rates in startle disease in animals.

In conclusion, we have identified a novel canine startle disorder with an autosomal recessive mode of inheritance, which is caused by the deletion of two coding exons from the GlyT2 gene, *SLC6A5*. The identification of the breakpoint and subsequent development of diagnostic PCRs has allowed the development of a diagnostic test (available via Laboklin http://www.laboklin.co.uk/). This has already enabled Irish wolfhound breeders to identify heterozygous animals (which have no discernable phenotypic differences when compared to wild-type animals), enabling rational breeding programs to be implemented that will limit further animal distress and premature death. This also highlights the growing compendium of animal models of startle disease that inform our knowledge of mechanisms of inhibitory neurotransmission as well as startle disease/hyperekplexia.

The following are the supplementary materials related to this article.

Gill et al. Supplementary Table 1 Primer sequences for canine *GLRA1*, *GLRB*, and *SLC6A5* exon amplification.

Gill et al. Supplementary Table 2 Probes for *SLC6A5* MLPA.

Supplementary Video 1

## Role of the funding source

The funders had no role in study design, data collection and analysis, decision to publish or preparation of the manuscript. None of the authors declare a conflict of interest.

## Figures and Tables

**Table 1:** Startle disorders in livestock and companion animals.

*Inherited congenital myoclonus (ICM) in Poll Hereford cattle*
Genetics: Autosomal recessive inheritance. Nonsense mutation (c.C156A, p.Y24X) in exon 2 of *GLRA1*, encoding the glycine receptor α1 subunit, resulting in a lack of synaptic α1βGlyRs. Clinical signs: Hyperesthesia (increased sensitivity to stimuli) and muscle rigidity, tremor, and myoclonic jerks of the skeletal musculature which occur spontaneously and in response to tactile, visual, or auditory stimuli. Survival of calves with ICM for up to ten days has been reported, but key independent activities required for survival, such as standing and feeding, are impaired. There are no detectable pathological lesions in the central nervous system, although compensatory mechanisms have been detected in affected animals including increases in presynaptic glycine uptake and GABAergic transmission. References: Harper et al., 1986; Gundlach, 1990; Gundlach et al., 1988; Lummis et al., 1990; Pierce et al., 2001.

*Congenital muscular dystonia type 2 (CMD2) in Belgian Blue cattle*
Genetics: Autosomal recessive inheritance. Missense mutation (c.C156A, p.Y24X; c.T809C, p.L270P) in exon 4 of *SLC6A5*, encoding the neuronal glycine transporter GlyT2, resulting in a loss of presynaptic glycine uptake and release. Clinical signs: Extreme muscle stiffness and tremor following acoustic, tactile, or visual stimulation. No overt pathological defects in the central nervous system. CMD2 homozygotes die within 24–48 h after birth due to respiratory difficulties. References: Charlier et al., 2008; Gill et al., 2011.
*Inherited myoclonus in Peruvian Paso horses*
Genetics: Likely autosomal recessive inheritance. Genetic lesion unknown, although likely to be a mutation in *GLRA1*, since [^^3^^H]strychnine binding sites are lost in the spinal cord ventral horn but retained in the dorsal horn, suggesting a loss of α1β but not α3β GlyRs. Clinical signs: From birth, myoclonic contractions of the skeletal musculature resulting from tactile or auditory stimuli. Additional evidence of hip pain, a characteristic “bunny hopping” gait with the hind limbs. If placed upright, animals are able to walk and run but in some cases are unable to rise alone. Reference: Gundlach et al., 1993.

*Familial reflex myoclonus in Labrador retriever dogs*
Genetics: Likely autosomal recessive inheritance. Genetic lesion unknown. Clinical signs: At six weeks old, three Labrador retrievers presented with muscle rigidity triggered by voluntary movements or handling, and respiratory distress was observed in severe episodes. Electromyogram analysis revealed increased motor unit amplitude with polyphasic action potentials without myotonic discharges. Normal serum chemistry, urinalysis, and muscle biopsies. Reference: Fox et al., 1984.
