# Supplementary material for: Startle disease in Irish wolfhounds associated with a microdeletion in the glycine transporter GlyT2 gene
Source: Neurobiol Dis. 2011 Jul;43(1):184–9. doi: 10.1016/j.nbd.2011.03.010 (PMC4068303; doi:10.1016/j.nbd.2011.03.010)
Supplement: Gill et al. Supplementary Table 1 — Primer sequences for canine GLRA1, GLRB, and SLC6A5 exon amplification. [file mmc1.doc]

**Gill et al Suppl. Table 2. Primer sequences for canine *GLRA1*, *GLRB* and *SLC6A5* exon amplification**

| **Gene** | **Exon** | **Forward Primer** | **Reverse primer** |
| --- | --- | --- | --- |
| ***GLRA1*** | 1 | aatatacccacccccaaacg | gagtcagcctcagtgctcct |
|  | 2 | ggcaaggctgggatatgat | atgtgtttgccatctgcgta |
|  | 3 | caagaggaatccatccctga | aactccatgcagagcgtaca |
|  | 4 | cgcagagcactgaatgaaag | tgggacaaggtgtgttctga |
|  | 5 | tgattttctggtaataactaaaatgg | gcagctgtatgggaatttctg |
|  | 6 | ccaaaccaattctgacatgct | ggtgggacctgaggaggata |
|  | 7 | cgctgaggttgttctgacct | taatgccttgtgctcccttt |
|  | 8 | ggggagttactgaggagcag | ggctgcttggaactcttctg |
|  | 9 | tagaggcagggagaaccaga | atgcatcactgcattttgct |
| ***GLRB*** | 1 | cattggatagtaatggtcatacgc | ttcgataacgcagtaacatttctc |
|  | 2 | tggtggctatttctaaatgctt | ttgttttgaatatcacaactaaacaat |
|  | 3 | gttttacatcctatgtggattgg | gcatccaccttagagttattcctta |
|  | 4 | tgtgctgcttatcactgaaactc | gggaaacaaggtcaagtcca |
|  | 5 | agcatgaggtactttttaatatttttg | caaaagcattagttttaccattgtt |
|  | 6 | tggcctgttatcttaccatttct | aaatatctgaacgcaaagataaacta |
|  | 7 | tgatttttatagttgggcgtagg | caaggaaaaggttcccatca |
|  | 8 | cttggaagagccttgacgag | ctgcacaccacggaaaagta |
|  | 9 | tcaggtaatctgatggcctaat | caatcaaatggcatccaaaa |
| ***SLC6A5*** | 1 | cctttaaaactggaatccaagg | gaaaggagcaccctaaagga |
|  | 2 | atccaccttccaggttattccctag | gctgcctcccgcatccatta |
|  | 3 | acactctcggtgcagggttga | tgccctctggcttagcactgat |
|  | 4 | gcttggtagaggagggggta | ccctgaattctgcagttcac |
|  | 5 | gagagacagtccgctccact | gagctctcgaaatgctagaactaa |
|  | 6 | ctctccaatatccggcaagt | gatgcaacccatcccttg |
|  | 7 | tgacctcagagggtccctaa | ttcggctcagggtgtgat |
|  | 8 | ggggaaactgccctgatg | ggtgcccaggtctcattaaa |
|  | 9 | agccagtaagttggtgctgaa | gctcggccttagcaggtg |
|  | 10 | gccacgtttatgcgtcct | ctctagccctgcctgagctt |
|  | 11 | ataaccaccgatggaagcag | caggcacctcaccatcctat |
|  | 12 | ttagagccatcaggggaatg | gggtgaaaggacagatgagc |
|  | 13 | tttgcttgcacctgactttc | agccaccttttgcttctcaa |
|  | 14 | tgtctgatggcttcttgcat | gggtgagtcacagctaagca |
|  | 15 | aagaaagcatccaagtcgtg | cacttaatcaccgcgtctcc |
|  | 16 | cagtttggggatcgatgg | catgcgacaccctatcttacttt |
