# Supplementary material for: Startle disease in Irish wolfhounds associated with a microdeletion in the glycine transporter GlyT2 gene
Source: Neurobiol Dis. 2011 Jul;43(1):184–9. doi: 10.1016/j.nbd.2011.03.010 (PMC4068303; doi:10.1016/j.nbd.2011.03.010)
Supplement: Gill et al. Supplementary Table 2 — Probes for SLC6A5 MLPA. [file mmc2.doc]

**Gill et al Suppl. Table 3. Probes for *SLC6A5* MLPA**

| **Probe** | **Sequence** | **Tm°C** | **%GC** | **Size** |
| --- | --- | --- | --- | --- |
| **CFTRa** | GGGTTCCCTAAGGGTTGGAGCTCCATTGCAATCTACCTAGCCATTG | 72 | 48 | 100 |
|  | P-GCTTATGCCTTCTCTTTATCATGAGGCCGCTTCTAGATTGGATCTTGCTGGCAC | 75 | 48 |  |
| **CFTRb** | GGGTTCCCTAAGGGTTGGAGCGCATTTCGTGTGGATTGCTC | 72 | 55 | 88 |
|  | P-CTCTACAAGTGACGCTCCTGATGGTCTAGATTGGATCTTGCTGGCAC | 70 | 54 |  |
| **SLC6A5**  **Exon 1** | GGGTTCCCTAAGGGTTGGAGAAGGGCACCTTGCTTTGCACTGAC | 73 | 54 | 87 |
|  | P-GCAAACCTGGCCTCTCCCAAGAAGGTCTAGATTGGATCTTGCTGGCAC | 73 | 62 |  |
| **SLC6A5**  **Intron2** | GGGTTCCCTAAGGGTTGGAGAGACCGGTTGGCAGTGCCAG | 75 | 67 | 85 |
|  | P-GTAATGGATGCGGGAGGCAGCTAGGAGCCCGAGGGGTGTCCTCTAGATTGGATCTTGCTGGCAC | 73 | 59 |  |
| **SLC6A5**  **Exon3** | GGGTTCCCTAAGGGTTGGAGAGTGGGGCTGGGCAATGTCTG | 72 | 62 | 85 |
|  | P-GAGGTTTCCCTACCTGGCCTTCAGGCTGGGGAAGAGCAAAATCACGGAGTCTCTAGATTGGATCTTGCTGGCAC | 71 | 59 |  |
| **SLC6A5**  **Exon4** | GGGTTCCCTAAGGGTTGGAGAAGTGTCACTGGGCCAGTTTGC | 70 | 55 | 85 |
|  | P-CAGCCAGGGGCCAGTGTCTGTGCTGGTCTAGTCTAGATTGGATCTTGCTGGCAC | 75 | 67 |  |
| **SLC6A5**  **Exon5** | GGGTTCCCTAAGGGTTGGAGCAATGCTGATCATCTCGGTCCTGATAG | 72 | 48 | 110 |
|  | P-CCATCTACTACAATGTGATTATCTGCTACACACTTTTCTACTCTAGATTGGATCTTGCTGGCAC | 72 | 37 |  |

Key: P- = 5' phosphate group, Blue: 5' PCR primer target, Green: 3' PCR primer target, Red = stuffer
