# Supplementary figures and images for: Startle disease in Irish wolfhounds associated with a microdeletion in the glycine transporter GlyT2 gene
Source: Neurobiol Dis. 2011 Jul;43(1):184–9. doi: 10.1016/j.nbd.2011.03.010 (PMC4068303; doi:10.1016/j.nbd.2011.03.010)

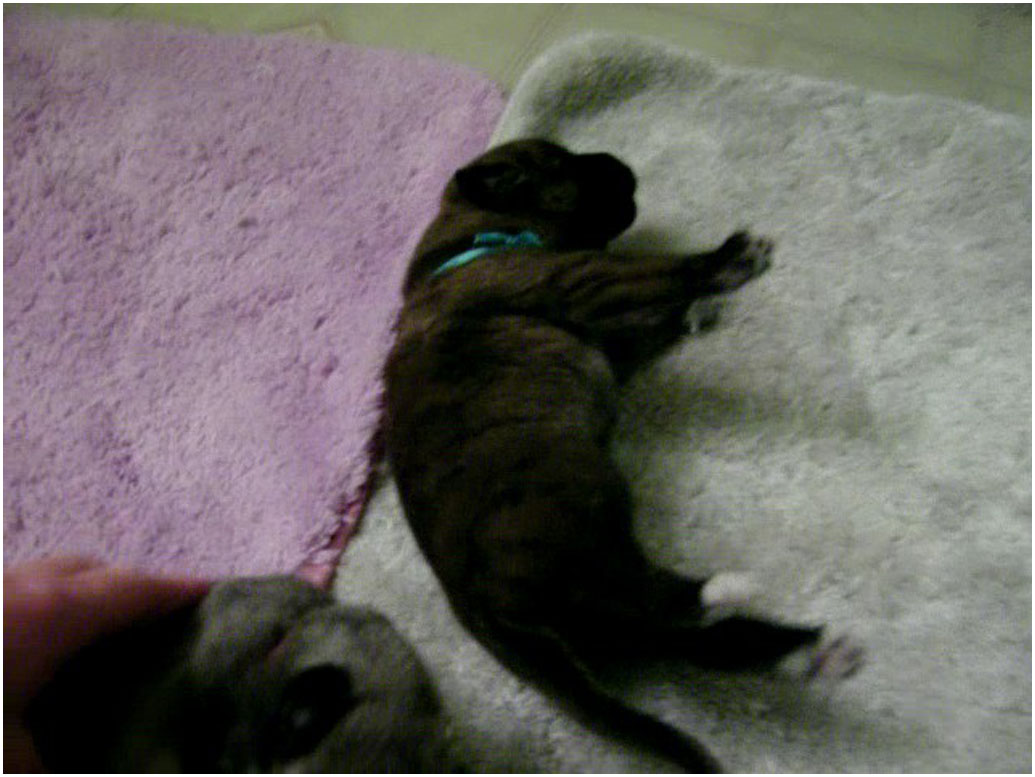

Supplement: Supplementary file 3 — Supplementary Video 1 [file mmc3.jpg]
